# Systematic Analysis of Neurotransmitter Receptors in Human Breast Cancer Reveals a Strong Association With Outcome and Uncovers HTR6 as a Survival-Associated Gene Potentially Regulating the Immune Microenvironment

**DOI:** 10.3389/fimmu.2022.756928

**Published:** 2022-03-10

**Authors:** Wei Zhang, Lintai Li, Jianxuan Li, Haiyan Yu, Fengping Zheng, Bin Yan, Wanxia Cai, Yumei Chen, Lianghong Yin, Donge Tang, Yong Xu, Yong Dai

**Affiliations:** ^1^ Department of Clinical Medical Research Center, The Second Clinical Medical College, Jinan University (Shenzhen People’s Hospital), Shenzhen, China; ^2^ South China Hospital, Health Science Center, Shenzhen University, Shenzhen, China; ^3^ HaploX Biotechnology, Shenzhen, China; ^4^ Department of Nephrology, Institute of Nephrology and Blood Purifification, The First Affifiliated Hospital of Jinan University, Jinan University, Guangzhou, China; ^5^ Shenzhen Second People’s Hospital, The First Affifiliated Hospital of Shenzhen University, Shenzhen, China

**Keywords:** neurotransmitter receptors, HTR6, breast cancer, immune microenvironment, antipsychotics

## Abstract

Many epidemiological reports have indicated an increase in the incidence of breast cancer among psychotic patients, suggesting that the targets of antipsychotics, neurotransmitter receptors, may have a role in tumorigenesis. However, the functions of neurotransmitter receptors in cancer are barely known. Here, we analyzed 44 neurotransmitter receptors in breast cancer and revealed that the expression of 34 receptors was positively correlated with relapse-free survival rates (RFS) of patients using the public database (n = 3951). Among all these receptors, we revealed decreased expression of HTR6 in human advanced breast cancer versus tumors *in situ* using our original data (n = 44). After a pan-cancer analysis including 22 cancers (n = 11262), we disclosed that HTR6 was expressed in 12 tumors and uncovered its influence on survival in seven tumors. Using multi-omics datasets from Linkedomics, we revealed a potential regulatory role of HTR6 in MAPK, JUN, and leukocyte-differentiation pathways through enriching 294 co-expressed phosphorylated proteins of HTR6. Furthermore, we proclaimed a close association of HTR6 expression with the immune microenvironment. Finally, we uncovered two possible reasons for HTR6 down-regulation in breast cancer, including deep deletion in the genome and the up-regulation of FOXA1 in breast cancer, which was a potential negatively regulatory transcription factor of HTR6. Taken together, we revealed a new function of neurotransmitter receptors in breast cancer and identified HTR6 as a survival-related gene potentially regulating the immune microenvironment. The findings in our study would improve our understanding of the pathogenesis of breast cancer and provided a theoretical basis for personalized medication in psychotic patients.

## Introduction

Lately, The World Health Organization’s International Agency for Research on Cancer (IARC) released the data on the global cancer burden in 2020 that breast cancer becomes the world’s most common cancer, surpassing lung cancer for the first time (1). Although the overall survival rate (OS) of breast cancer has been significantly improved in recent years, breast cancer still brings great harm to patients, including physical burdens and side effects after drug treatment. At the same time, the experience of breast cancer takes a great toll on a woman physically (image damage caused by surgery and treatment) and psychologically (2, 3).

Many studies have shown that the incidence of breast cancer in psychiatric patients is significantly higher than the normal population (4–7). Food and Drug Administration (FDA) has proclaimed that antipsychotics are potentially tumorigenic (8). Besides, many pieces of research have demonstrated that the use of antipsychotics is associated with an increased risk of breast cancer (9). Until 2019, the number of people with mental illness has reached 1.55 billion, or 22.1% of the global population (10, 11). Many patients with mental illness need to take medicine “for a long time” or even “for the whole life”; thus, the potential tumorigenicity of antipsychotics puts numerous psychotic patients at potential cancer risk.

Some studies have shown that the increase of prolactin may be associated with antipsychotics-related breast cancer (9). However, the mechanism underlying the potential tumorigenicity of antipsychotics is still unclear. Psychiatric medications usually block the dopamine system or simultaneously block the dopamine and the serotonin systems (12). Other common targets of psychiatric drugs include cholinergic receptors and adrenergic receptors, etc. (12). Recent research has provided clues that genes commonly targeted by psychiatric drugs (neurotransmitter receptors) may play a role in the development of cancer (13–19), implying the potential oncogenic properties of antipsychotics may be related to the activity changes of these neurotransmitter receptors after drug administration. However, there are still very few studies disclosing the functions of neurotransmitter receptors in tumorigenesis.

In this study, we investigated four families of neurotransmitter receptors commonly targeted by antipsychotics, including dopamine receptors, serotonin receptors, cholinergic receptors, and adrenergic receptors (44 receptors in total). As a result, the expression of 34 genes showed a positive correlation with relapse-free survival rates (RFS) of breast cancer patients, suggesting that neurotransmitter receptors were likely to be suppressors involved in the development of tumors. After a filter screening, we selected HTR6 for a further in-depth study. Firstly, we validated HTR6 expression in breast tumors using our original data (n = 44). Next, we explored the function of HTR6 in different subtypes of breast cancer by analyzing its influence on patient survival. Furthermore, we uncovered the correlation between HTR6 expression and the immune microenvironment. Finally, we depicted the pathways that HTR6 participated in and revealed the reasons for the altered expression of HTR6 in breast cancer using multi-omics analyses.

## Material and Methods

### Patients

The paraffin blocks of breast cancer tissue were provided by the National Human Genetic Resources Sharing Service Platform. Patients who had breast tissue surgically harvested ranged in age from 30 to 89, with an average age of 58. Patients with breast carcinoma in situ, breast invasive ductal carcinoma, and metastatic breast cancer were included in this study. All patients were informed and signed the informed consent. The work was carried out under the supervision of the Ethics Committee of Taizhou Hospital, in line with the requirements of the World Medical Association for the execution of human experiments.

### Immunohistochemical Assay

The tissue chips were left at room temperature for 60 minutes and then soaked in xylene for 10 minutes. The soaking step was repeated once more. Next, the chips were dewaxed in 100%, 95%, and 75% ethanol, respectively, and heated at 95°C in sodium Lycium buffer (pH = 6.0, 0.01 M) for 15 min. This heating step was repeated 1-2 times. The chips were then cleaned with PBS solution three times and 5 minutes for each time. Subsequently, the chips were dropped onto 3% H2O2, standing at room temperature for 10 minutes, and cleaned with PBS solution three times and 5 minutes each time. Subsequently, the chips were incubated with blocking solution at room temperature for 20 minutes. And then, the primary antibody of HTR6 was added onto the chip (Creative Diagnostic, DCABH-15695; 1:200), and the chips were incubated at 4°C for 12 h. The chips were cleaned with PBS solution three times and 5 minutes for each time. Then the incubation solution with secondary antibody was added onto the chip, and the chips were incubated at 37°C for 1 h. Next, the chips were cleaned with PBS solution three times and 5 minutes for each time. DAB was used to color the chips, and the staining was observed under a microscope. Subsequently, the chips were rinsed with running water for 10 minutes, incubated with hematoxylin for 2 min, and differentiated with hydrochloric acid alcohol. After being washed with tap water for 10 minutes, the chips were dehydrated, transparent, and sealed. Next, the cell staining was photographed by Aperio scanner (LEICA, Aperio XT). The staining intensity and proportion were assessed by two pathologists. Staining score was calculated by multiplying staining intensity by staining proportion.

### Kaplan-Meier Plotter

Kaplan-meier Plotter (20) is a comprehensive database that summarizes RNA-sequencing and gene-array data sets from The Cancer Genome Atlas (TCGA), Gene Expression Omnibus (GEO) and other databases. All subtypes of breast cancer were included in the analysis. The effects of HTR6 expression on the RFS of breast cancer patients were analyzed through the “Breast cancer” module in the “Gene Chip” column. The influence of HTR6 expression on the overall survival rates (OS) of patients with various tumors were analyzed through the “Pan-cancer” module in the “RNA-Seq” column.

### Linkedomics

Linkedomics (21) collects together a variety of omics data sets from TCGA, Clinical Proteomic Tumor Analysis Consortium (CPTAC) and multiple other databases, enabling online multi-omics joint analysis and visualization. The exploration of HTR6 co-expressed phosphorylation was performed using the data sets from Linkedomics.

### The Human Protein Atlas

The Human Protein Atlas provides data on the protein expression of different tissues and tumors. The expression of the FOXA1 protein in breast cancer and normal tissue was analyzed using The Human Protein Atlas.

### cBioPortal

The database included over 28,000 samples from various genomic studies. All independent studies on breast cancer were included in our analysis. The frequencies and types of HTR6 mutations were analyzed using the modules “Oncoprint” and “Cancer Types Summary”.

### Human Transcription Factor Targets (hTFtarget)

The hTFtarget database has collected over 7,000 ChIP-Seq samples and included over 600 experimentally-confirmed transcription factors. This study focused on the HTR6 upstream transcription factors (TFs) in breast tissues.

### CIBERSORT and XCELL

CIBERSORT (22) and XCELL (23) are algorithms determining the probable proportions of immune cells in samples based on gene expression profiles. In this study, the algorithms CIBERSORT and XCELL were used to characterize the composition of the immune cells.

### Statistical Analyses

The log-rank method was utilized to analyze the significance of the expression of a gene on patient survival. Fisher’s exact test was used to analyze the significance of enrichment. A p-value of less than 0.05 was regarded as significant. ***p < 0.001, **p < 0.01, and *p < 0.05.

## Results

### The Expression of Most Neurotransmitter Receptors Was Positively Associated With the RFS of Breast Cancer and HTR6 Was One Potential Functional Gene in Tumorigenesis

Common targets of psychiatric drugs include neurotransmitter receptors of dopamine receptors, serotonin receptors, cholinergic receptors, adrenergic receptors, and glutamate receptors. In this study, we performed RFS analyses on dopamine receptors, serotonin receptors, adrenergic receptors, and cholinergic receptors (44 neurotransmitter receptors) in breast cancer patients (n = 3951) using the Kaplan-Meier Plotter database. Surprisingly, the expression of 34 genes was positively correlated with RFS (the remaining ten receptors were not significantly correlated with RFS), suggesting that these genes might be tumor suppressors in breast cancer (**Supplementary Figures 1–4**, **Table 1**). Subsequently, we performed further screening in these 34 genes to identify the functional genes. Because epidemiological reports have indicated that the incidence of lung cancer among patients with mental illness has decreased (24, 25), we speculated that the expression of functional genes may be negatively correlated with the RFS of patients with lung cancer. Therefore, we investigated the association between the expression of these 44 receptors and the RFS of lung cancer patients (n = 1925). As a result, 21 receptor expression was negatively correlated with the survival of patients with lung cancer, five receptors were positively correlated, and 18 receptor expression was not significantly correlated with the survival of patients with lung cancer (**Supplementary Figures 5–8**). This result further confirmed that neurotransmitters might be related to the development of tumors.

**Table 1:** The analysis of the expression of 44 neurotransmitter receptors on RFS of breast cancer (n = 3951).

NO.	Receptors	RFS
1	HTR1A	Positive
2	HTR1B	Positive
3	HTR1E	Positive
4	HTR2A	None
5	HTR2B	None
6	HTR2C	Positive
7	HTR3A	Positive
8	HTR3B	None
9	HTR3C	Positive
10	HTR4	Positive
11	HTR5A	Positive
12	HTR6	Positive
13	HTR7	Positive
14	DRD1	Positive
15	DRD2	Positive
16	DRD3	Positive
17	DRD4	Positive
18	DRD5	Positive
19	ADRB1	None
20	ADRB2	Positive
21	ADRB3	Positive
22	ADRA1B	Positive
23	ADRA1D	Positive
24	ADRA2A	Positive
25	ADRA2B	Positive
26	ADRA2C	None
27	CHRM3	None
28	CHRM2	Positive
29	CHRNE	Positive
30	CHRM1	None
31	CHRNA7	None
32	CHRND	Positive
33	CHRNA1	Positive
34	CHRNB2	None
35	CHRM4	Positive
36	CHRM5	Positive
37	CHRNA3	Positive
38	CHRNB4	Positive
39	CHRNB1	Positive
40	CHRNA2	Positive
41	CHRNA5	None
42	CHRNG	Positive
43	CHRNB3	Positive
44	CHRNA6	Positive

Next, we overlapped the receptors screened out by survival analyses in breast cancer and lung cancer and obtained 20 genes that have an influence on patient survival in both breast cancer and lung cancer for further study (**Table 2**). Based on the fact that genes that had been proven to be related to the progression of breast cancer including DRD1 and HTR7 were both Gsα protein-coupled receptors (GsαPCRs) (26, 27), and several studies had shown that Gsα protein was associated with malignant transformation of a variety of tumors (28–30), we further filtered the 20 candidate receptors and obtained three GsαPCRs, including DRD1, DRD5, and HTR6. DRD1 is a frequently activated target of psychiatric drugs, but we discovered that the low expression of DRD1 was correlated with poor RFS of breast cancer in the above study, so DRD1 might be not involved in the potential tumorigenicity of psychiatric drugs. Therefore, we selected DRD5 and HTR6 for the following analysis. We summarized 21 common antipsychotic drugs and found that HTR6 was highly compatible with 85.7% of drugs (18 drugs) (**Table 3**), suggesting it was a high-frequency target of antipsychotic drugs. Therefore, we selected HTR6 for further study.

**Table 2:** The 20 candidate receptors associating with survival of both breast cancer and lung cancer.

NO.	Receptors	Breast cancer	Lung cancer	Coupled G protein
1	HTR1B	Positive	Negative	Gi
2	HTR2C	Positive	Negative	Gi
3	HTR3A	Positive	Negative	Gq
4	HTR3C	Positive	Negative	Gq
5	HTR6	Positive	Negative	Gsα
6	DRD1	Positive	Negative	Gsα
7	DRD2	Positive	Negative	Gi
8	DRD3	Positive	Negative	Gi
9	DRD4	Positive	Negative	Gi
10	DRD5	Positive	Negative	Gsα
11	ADRA2B	Positive	Negative	Gi
12	CHRM2	Positive	Negative	Not clear
13	CHRM4	Positive	Negative	Gi
14	CHRM5	Positive	Negative	Not clear
15	CHRNA3	Positive	Negative	Not clear
16	CHRNB4	Positive	Negative	Not clear
17	CHRNB1	Positive	Negative	Not clear
18	CHRNG	Positive	Negative	Not clear
19	CHRNB3	Positive	Negative	Not clear
20	CHRNA6	Positive	Negative	Not clear

**Table 3:** The antipsychotic drugs targeting DRD5 or HTR6.

NO.	Drugbank ID	Name	Binding
1	DB01624	Zuclopenthixol	DRD5
2	DB01038	Carphenazine	DRD5
3	DB00246	Ziprasidone	DRD5\HTR6
4	DB01238	Aripiprazole	DRD5\HTR6
5	DB00334	Olanzapine	DRD5\HTR6
6	DB01224	Quetiapine	DRD5\HTR6
7	DB01403	Methotrimeprazine	DRD5
8	DB00477	Chlorpromazine	DRD5\HTR6
9	DB00408	Loxapine	DRD5\HTR6
10	DB14185	Aripiprazole lauroxil	DRD5\HTR6
11	DB06216	Asenapine	HTR6
12	DB00363	Clozapine	HTR6
13	DB04946	Iloperidone	HTR6
14	DB00458	Imipramine	HTR6
15	DB00543	Amoxapine	HTR6
16	DB01142	Doxepin	HTR6
17	DB00321	Amitriptyline	HTR6
18	DB06148	Mianserin	HTR6
19	DB09225	Zotepine	HTR6
20	DB00502	Haloperidol	HTR6
21	DB06144	Sertindole	HTR6

### The Expression of HTR6 Was Down-Regulated in Advanced Breast Cancer Versus Breast Cancer *In Situ*

**Figure 1:**
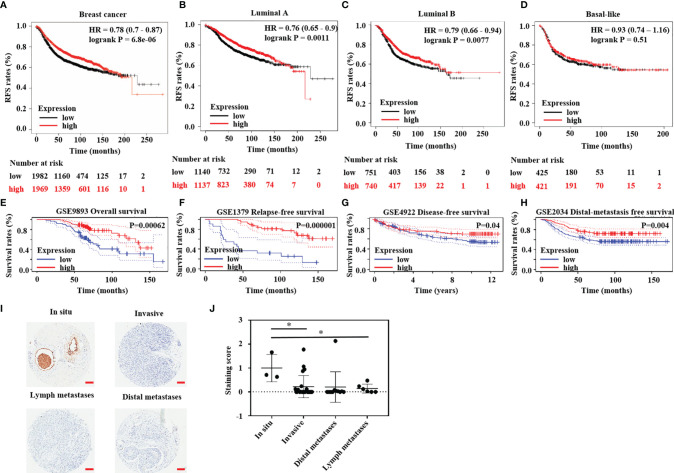
HTR6 had a potential role in human breast cancer. **(A)** Lower expression of HTR6 was correlated with lower RFS in breast cancer patients (n = 3951); Lower expression of HTR6 was correlated with **(B)** lower RFS in patients with Luminal A breast cancer and **(C)** Luminal B breast cancer; But not in **(D)** patients with Basal-like breast cancer. **(E–H)** Lower expression of HTR6 was correlated with lower OS, RFS, distal metastasis free survival rate, and disease free survival rate in breast cancer patients. **(I, J)** The expression of HTR6 was reduced in invasive breast cancer and metastases versus breast cancer in situ, and “*” indicated p value < 0.05.

In the above studies, we found that HTR6 expression was positively correlated with the RFS of breast cancer (**Figure 1A**). Subsequently, we analyzed the RFS of different subtypes of breast cancer responding to HTR6 expression. As a result, HTR6 expression was associated with the RFS of Luminal A and Luminal B breast cancer, but not Basal-like (**Figures 1B–D**). Subsequently, we analyzed the effect of HTR6 expression on the OS and other types of survival rates using the data sets from Kaplan-Meier Plotter database, and we did not obtain statistically significant conclusions. This might be because of a variety of factors, such as the size of the cohort, race, and breast cancer subtype. To confirm that HTR6 expression affected the patient survival rates, we performed further analysis using the data sets from the Prognoscan, and uncovered that HTR6 expression was statistically correlated with the OS, RFS, distal metastasis free survival rate, and disease free survival rate (P < 0.05) (**Figures 1E–H**). To verify the expression of HTR6 in breast cancer and explore its functions in tumor progression, we detected HTR6 protein in breast cancer tissues from 44 patients (**Table 4**) using an immunohistochemical assay. We found that the expression of HTR6 in invasive breast cancer, lymph node metastases, and distal metastases was lower than that *in situ* breast cancer (**Figures 1I, J**). These results supported what we found above and indicated that HTR6 might have an inhibitory effect on breast cancer progression (invasion, metastasis, etc.)

**Table 4:** The characteristics of 44 breast cancer patients in our study.

	Type	Patients
**Age**	≤65	29 (65.9%)
	>65	15 (34.1%)
**Sex**	Female	44 (100%)
**Subtypes**	Carcinoma *in situ*	3 (6.8%)
	Invasive ductal carcinoma	29 (65.9%)
	Invasive ductal carcinoma with mucinous adenocarcinoma	1 (2.3%)
	Metastatic carcinoma	11 (25.0%)
**Staging**	0	3 (6.8%)
	1	4 (9.1%)
	2	10 (22.7%)
	3	7 (15.9%)
	4	14 (31.8%)
	–	6 (13.7%)
**T-stage**	Tis	3 (6.8%)
	T1	5 (11.4%)
	T2	20 (45.5%)
	T3	2 (4.5%)
	T4	0 (0%)
	–	14 (31.8%)
**M-stage**	M0	30 (68.2%)
	M1	14 (31.8%)
**N-stage**	N0	11 (25.0%)
	N1	6 (13.6%)
	N2	4 (9.1%)
	N3	3 (6.8%)
	–	20 (45.5%)

Tis: High grade dysplasia;

“–”: The staging and T-stage of patients with distal or lymphatic metastases was not identified.

### HTR6 Was Potentially Involved in the Development of Multiple Tumors

**Figure 2:**
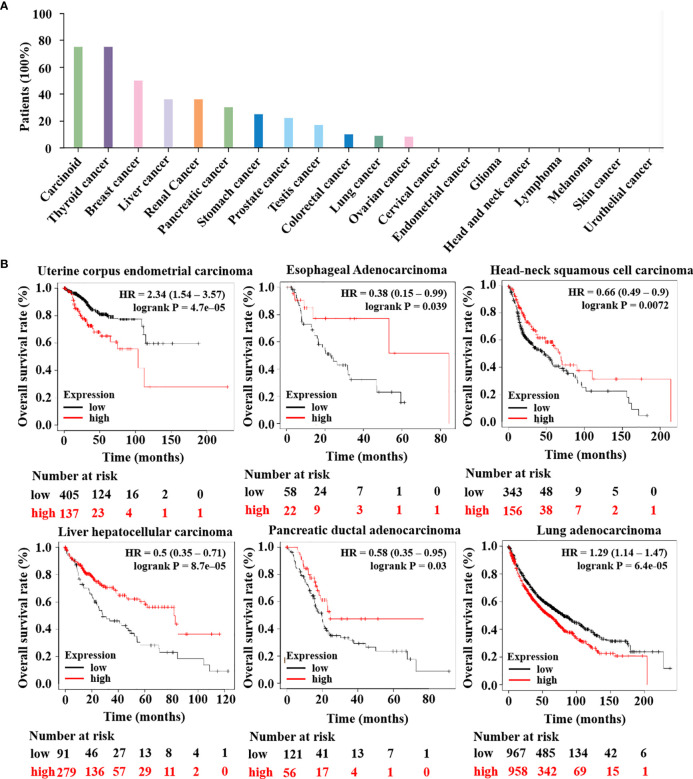
The expression of HTR6 was associated with the OS of a variety of tumors. **(A)** HTR6 protein was expressed in many tumors, including breast cancer. **(B)** The expression of HTR6 was associated with the OS of multiple tumors.

Until now, most research on HTR6 has focused on its role in the nervous system (31–33). Subsequently, to solid the role of HTR6 in tumorigenesis, we primarily analyzed the protein expression of HTR6 in 20 kinds of tumors using The Human Protein Altas database. The results showed that HTR6 was universally expressed in benign tumors, thyroid cancer, breast cancer, and other nine human tumors (**Figure 2A**). Moreover, we analyzed the association between HTR6 expression and the OS of 21 types of tumors (n = 11262) using the Kaplan-Meier Plotter database. As a result, HTR6 expression was positively correlated with the OS of esophageal adenocarcinoma (n = 80), head and neck squamous cell carcinoma (n = 500), hepatocellular carcinoma (n = 371), and ductal adenocarcinoma of the prostate (n = 177), and negatively correlated with lung cancer (n = 1925) and endometrial carcinoma (n = 543) (**Figure 2B**). These results revealed that HTR6 might be correlated with the initiation and development of a variety of tumors.

### The Functions of HTR6 in Breast Cancer Might Be Associated MAPK, JUN, and Immune Pathways

**Figure 3:**
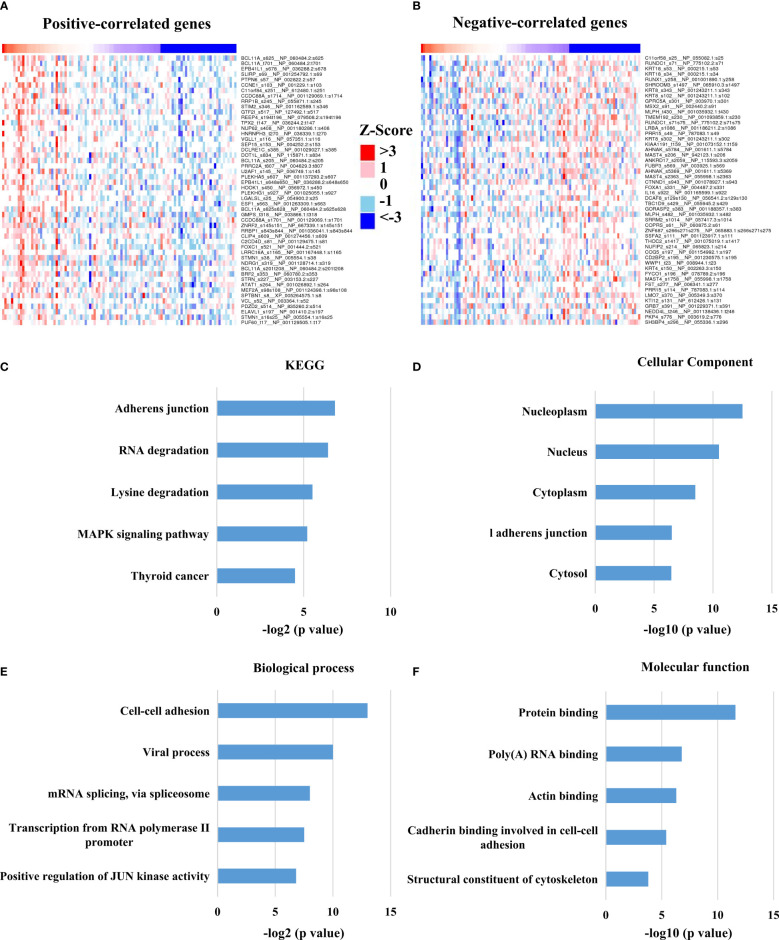
HTR6 was potentially involved in the regulation of MAPK, JUN and immune pathways in breast cancer. **(A)** The top 50 phosphorylation proteins positively correlated with HTR6 mRNA; **(B)** The top 50 phosphorylation proteins negatively correlated with HTR6 mRNA. 294 HTR6 co-expressed phosphorylated proteins (correlation coefficient > 0.3 or < -0.3) were enriched by **(C)** KEGG, **(D)** Cellular component, **(E)** Biological process, and **(F)** Molecular function.

At present, the role of HTR6 in tumors and the underlying mechanism are still barely known. In the above study, we found that HTR6 influenced the RFS of breast cancer; thus, we further explored the pathways or genes involved in this process. We analyzed the biological processes and pathways that HTR6 might regulate in breast cancer. Since HTR6 is a GsαPCRs, the activation of HTR6 triggers the activity of Gsα protein, thus promoting the generation of cAMP (34). As the downstream effector of cAMP, Cyclic AMP-dependent protein kinase A (PKA) phosphorylates many substrates (35). Therefore, we analyzed the phosphorylation modifications co-expressed with HTR6 through the Linkomics database (21). As a result, the phosphorylation of 294 genes was correlated with HTR6 expression in breast cancer (correlation coefficient > 0.3 or < -0.3) (**Figures 3A, B**, **Supplementary Table**). KEGG enrichment analysis of the HTR6 co-expressed phosphorylations revealed that HTR6 might regulate the MAPK pathway and tumor-related pathway (**Figure 3C**). Meanwhile, GO enrichment showed that the changes of HTR6 expression might affect the processes including JUN kinase activity, mRNA splicing, gene transcription, and cytoskeleton (**Figures 3D–F**).

**Figure 4:**
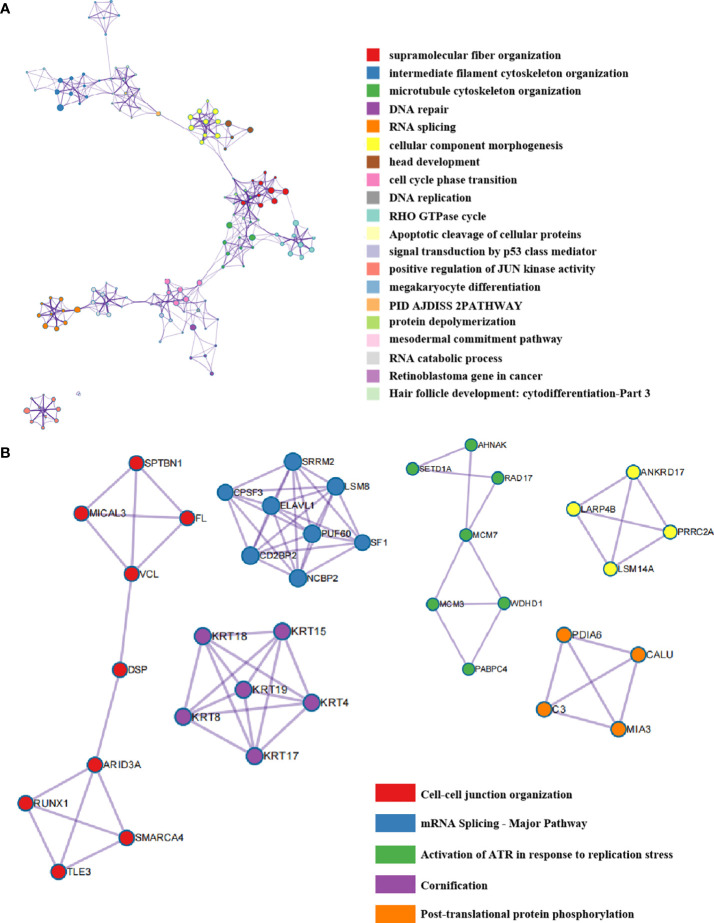
The interaction network of HTR6 co-expressed phorsphorylated proteins and key protein populations. **(A)** The protein interaction network of HTR6 co-expressed phorsphorylated proteins. **(B)** The closely-tied groups in the interaction network.

Moreover, we analyzed the enriched pathways of the co-expressed phosphorylated proteins using the Metascape database and discovered that the pathway named megakaryocyte differentiation was enriched. This finding suggested that HTR6 might be relevant to regulating the immune microenvironment in breast cancer (**Figure 4A**). In addition, we also constructed the interacting network of these biological processes (**Figure 4A**). We uncovered tightly-linked groups of these co-expressed proteins, including cell-cell junction organization, mRNA splicing-major pathway, and activation of ATR in response to replication stress, cornification, and post-translational protein phosphorylation (**Figure 4B**).

### HTR6 Potentially Regulated the Immune Microenvironment of Breast Cancer

**Figure 5:**
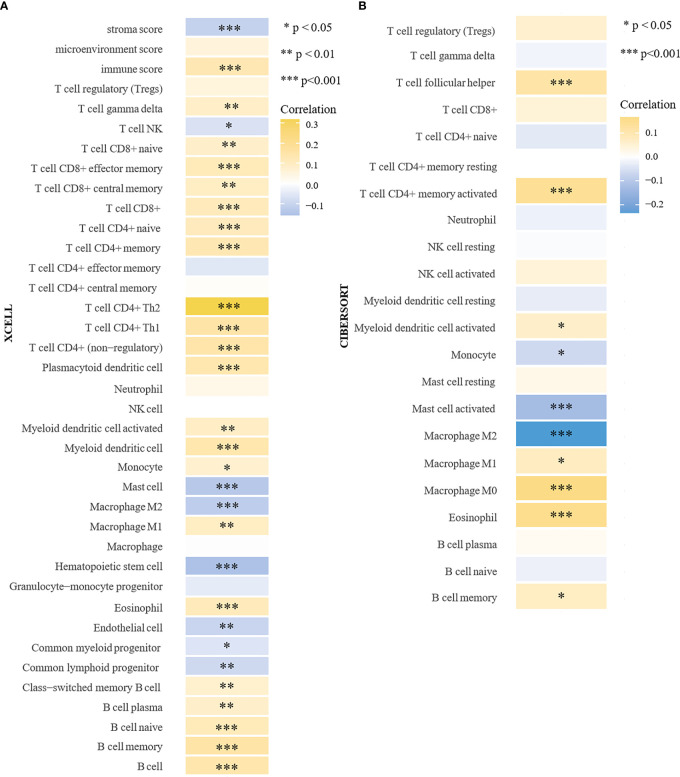
HTR6 expression was correlated with the infiltration of immune cells in breast cancer. **(A)** The analyses using Xcell and **(B)** CIBERSORT algorithms revealed the correlation between HTR6 expression and stroma score, immune score, and various types of immune cell infiltration in breast cancer.

**Figure 6:**
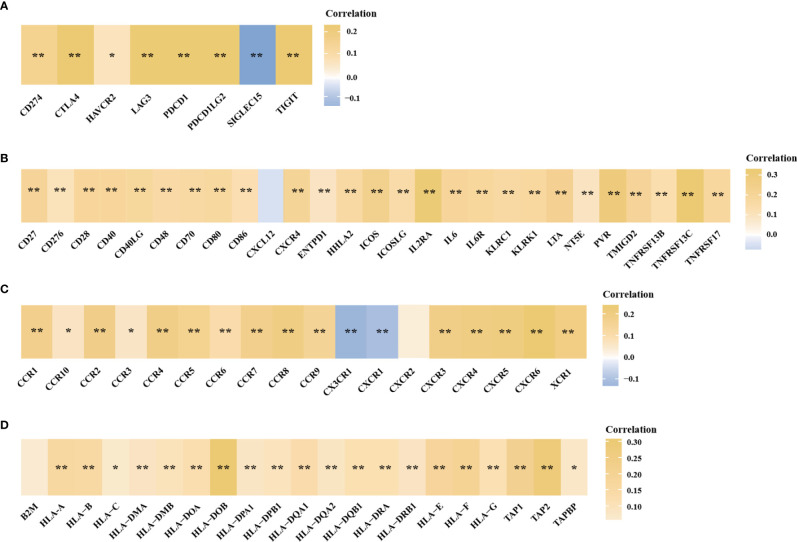
The expression of HTR6 was significantly correlated with the expression of immune checkpoints, immune stimulators, immune receptors and chemokines. The correlation between HTR6 expression and the expression of **(A)** immune checkpoints, **(B)** immune stimulators, **(C)** immune receptors, and **(D)** chemokines. “*” indicated p value < 0.05, and “**” indicated p value < 0.01.

In the above studies, we found that HTR6 might have a regulatory effect on immune-related pathways. Besides, MAPK and JUN pathways, which are also potentially regulated by HTR6, are closely associated with the immune microenvironment of tumors (36, 37). Therefore, we explored the role of HTR6 in the immune microenvironment of breast cancer. Using Xcell (23) and CIBERSORT algorithms (22), we analyzed the correlation between HTR6 expression and microenvironment scores. The results showed that HTR6 was strongly correlated with stroma score and immune score in breast cancer (p < 0.001) (**Figure 5A**). In addition, the expression of HTR6 was strongly correlated with the infiltration of many immune cells, such as CD4+ Th2 T cells, CD4+ memory T cells, and Macrophage M2 cells (**Figures 5A, B**). Furthermore, we also analyzed the correlation between the expression of HTR6 and the checkpoint genes, immune stimulators, immune receptors, and chemokine. As a result, HTR6 was significantly correlated with the expression of most immune checkpoints, chemokines, immune receptors, and immune stimulators (**Figures 6A–D**).

**Figure 7:**
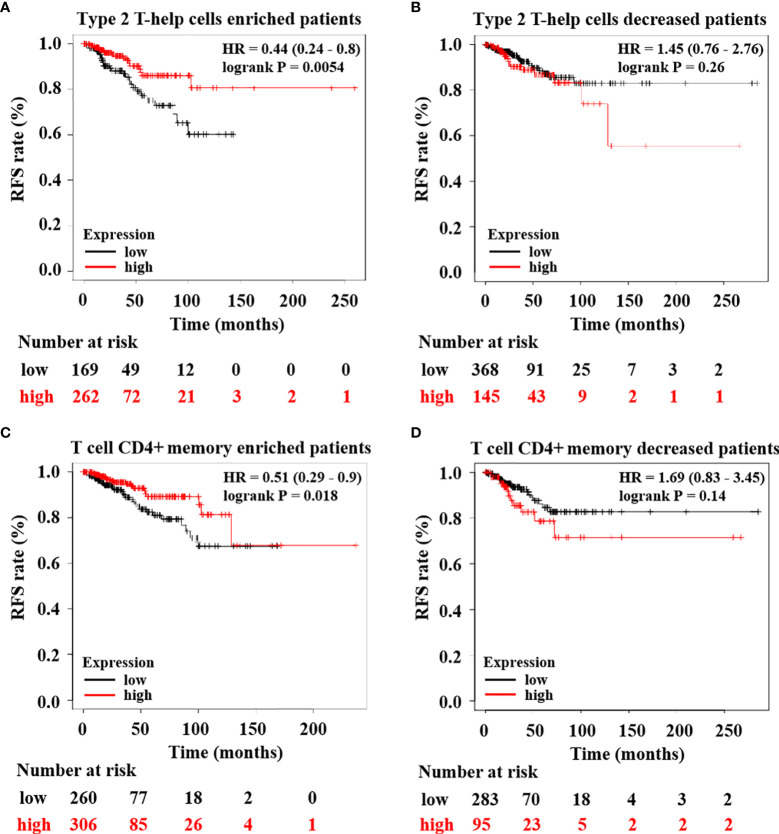
HTR6 expression was significantly correlated with the RFS of breast cancer patients with high immune infiltration. HTR6 expression was correlated with the RFS of breast cancer patients with **(A)** enriched type 2 T-help cells, but not correlated with the RFS of breast cancer patients with **(B)** decreased type 2 T-help cells. HTR6 expression was correlated with the RFS of breast cancer patients with **(C)** enriched CD4+ memory T cells, but not correlated with the RFS of breast cancer patients with **(D)** decreased CD4+ memory T cells.

Subsequently, to confirm HTR6 expression regulating immune infiltration and then affecting the development of breast cancer, we examined the correlation between HTR6 mRNA expression and RFS of breast cancer patients with high or low immune infiltration. The results showed that in patients with high type 2 T-help cells infiltration, HTR6 expression was positively correlated with RFS, while there was no significant effect on RFS in patients with low infiltration (**Figures 7A, B**). In patients with high infiltration of CD4+ memory T cells, HTR6 expression was positively correlated with RFS as well, while there was no significant effect in patients with low infiltration (**Figures 7C, D**). These results indicated that HTR6 expression was closely related to the immune microenvironment of breast cancer and thus affected the survival of patients.

### FOXA1 Was a Potential Negatively Regulatory TF of HTR6 in Breast Cancer

**Figure 8:**
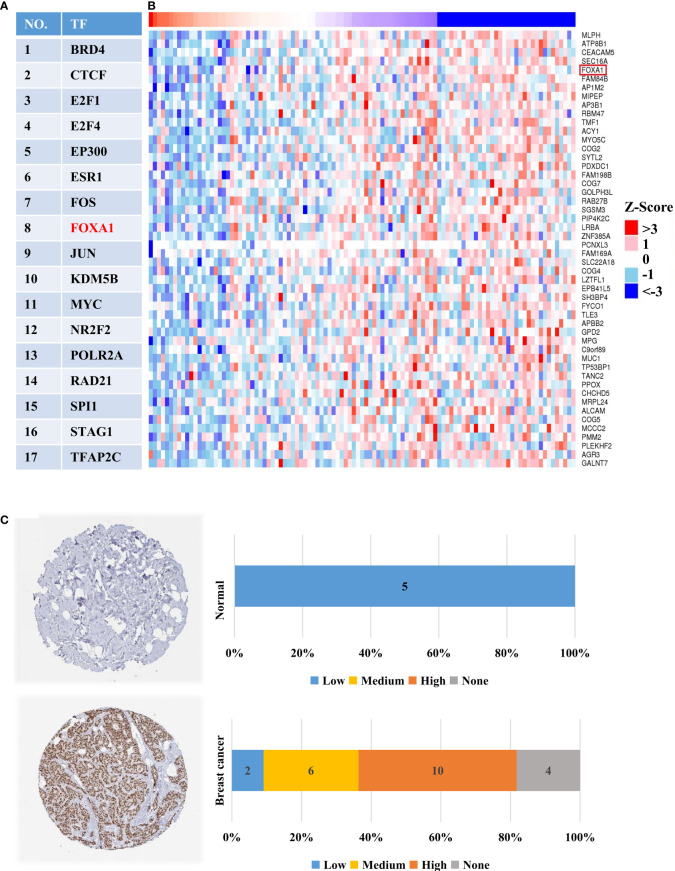
FOXA1 was one potential TF negatively regulating the expression of HTR6 in breast cancer. **(A)** 17 experimentally-tested TFs of HTR6 in breast tissue were uncovered using hTFtarget database. **(B)** FOXA1, as one potential TF of HTR6 in breast cancer was negatively correlated with HTR6 expression. **(C)** The expression of FOXA1 was up-regulated in most patients with breast cancer.

As is well-known, the expression of target genes is closely related to their upstream TF’s protein level. Consequently, we investigated the TFs of HTR6 using the hTFtarget database and discovered 17 experimentally tested TFs of HTR6 in breast tissue (**Figure 8A**). Meanwhile, through the Linkomics database, we disclosed that 181 proteins were co-expressed with HTR6 (correlation coefficient > 0.3 or < -0.3). After overlapping the 17 TFs and the 181 genes, we found that FOXA1, one of the HTR6 potential TF in breast cancer, was negatively correlated with HTR6 mRNA (correlation coefficient = -0.428) (**Figure 8B**). This suggested that FOXA1 might be one negative TF for HTR6 in breast cancer. Subsequently, we analyzed the expression of FOXA1 using the Human Protein Atlas database and observed that FOXA1 was up-regulated in more than 70% of patients with breast cancer (n _normal_ = 5, n _tumor_ = 22) (**Figure 8C**). Since HTR6 was down-regulated in invasive breast cancer and metastases versus tumor in situ, this result suggested that FOXA1 might be the negatively regulatory TF of HTR6 in breast cancer.

### Deep Deletion on the Genome Might Be Another Reason for the Down-Regulation of HTR6 in Breast Cancer

**Figure 9:**
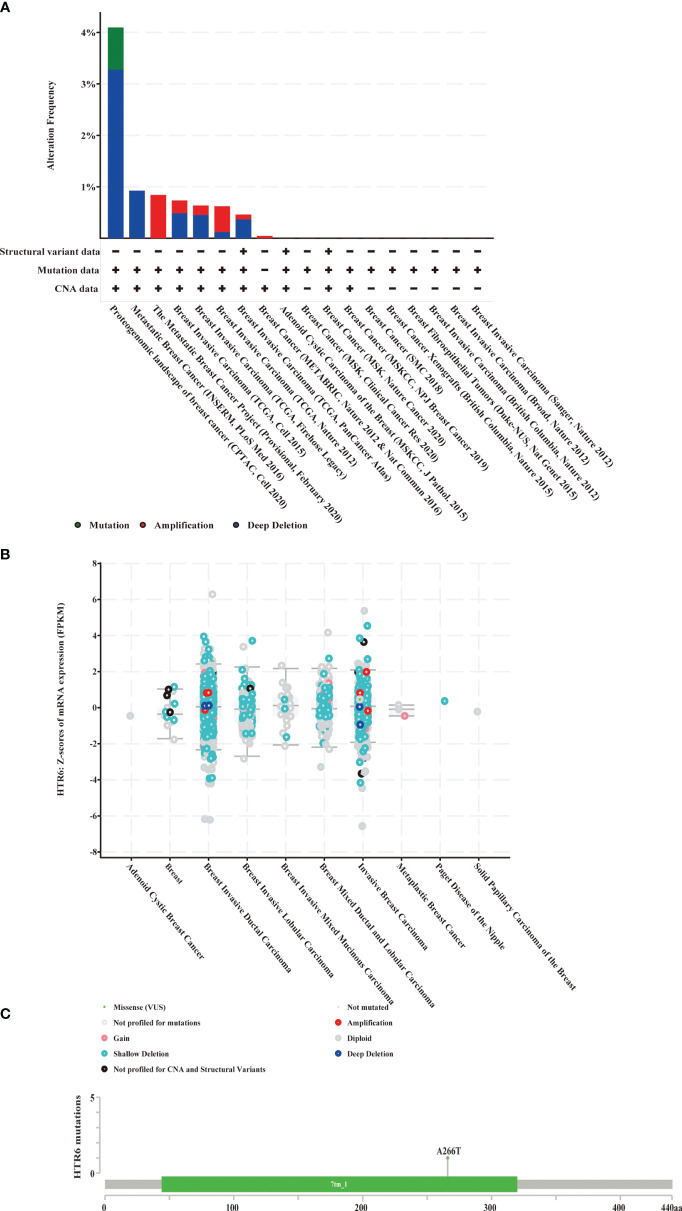
Deep deletion of HTR6 in breast cancer was possibly relevant to the down-regulation of HTR6. **(A)** The distribution of HTR6 mutations in different breast cancer studies. **(B)** The correlation between HTR6 mutations and expression. **(C)** The mutation site of HTR6 in breast cancer patients.

In the above studies, we found that FOXA1, as a negative regulatory TF of HTR6, was up-regulated in breast cancer, which might be one of the reasons why HTR6 was down-regulated. Subsequently, we collected DNA mutations of HTR6 found by independent studies of breast cancer using the Cbioportal database (n = 9555) (**Figures 9A, C**) and revealed that deep deletion (shallow deletion refers to the slight loss of copy number, which can be understood as the original diploid becomes the monoploid (generally defined by the value of log2Ratio). Deep deletion indicates a massive loss of copy number) covered more than 55% of all patients with HTR6 mutations. A study of metastatic breast cancer in 2016 showed a 100% frequency of deep deletion in the patients carrying HTR6 mutations (**Figure 9A**). Furthermore, to explore the relationship between deep deletion and HTR6 expression, we analyzed 3143 patients with mutations. We observed that the expression of HTR6 in patients with deep deletion was equal to or lower than the median expression of the whole mutated population (**Figure 9B**). Therefore, we speculated that deep deletion might be another reason for the down-regulation of HTR6 expression.

## Discussion and Conclusions

This study explored the role of 44 neurotransmitter receptors in breast cancer by the bioinformatics method and demonstrated that the expression of 34 receptors was positively correlated with the RFS of patients. Among all these receptors, we performed a further in-depth analysis of HTR6, uncovering its new function in tumorigenesis, especially in breast cancer, revealing a high correlation between the expression of HTR6 and immune microenvironment, disclosing the pathways and upstream regulators that might mediate HTR6 functions.

According to a report in 2019, the prevalence of mental diseases worldwide has reached 22.1% (that is, 1.547 billion people) (10). Epidemiological reports have shown an increased incidence of breast cancer in patients with psychiatric disorders and an increased risk of breast cancer in people taking antipsychotic medications (4). Because antipsychotic drugs can reduce symptoms in patients with mental illness and some patients need to take them “for a long time”, a large number of patients are at potential risk of developing cancer. In addition, some patients with severe mental illness lack intact cognition, perception, and communication and cannot be aware of the disease as soon as possible and seek medical treatment on their own. Therefore, tumor prevention in patients with mental illness is particularly important. However, the molecules mediating the potential carcinogenicity of psychiatric drugs remain poorly understood. Neurotransmitter receptors, as frequent targets of psychiatric drugs, have been uncovered being relevant to tumorigenesis (13–19). Therefore, we hypothesized that the potential oncogenicity of psychotic drugs might be mediated by their targeted neurotransmitter receptors. In this study, we revealed neurotransmitter receptors, were potentially associated with tumorigenicity of breast cancer and lung adenocarcinoma. The results are expected to be applied in tumor prevention, the design of antipsychotic drugs, and the personalization of clinical psychotherapy.

Neurotransmitter receptors are widely expressed in the central nervous system (CNS) and play an important role in transmitting neurotransmitters between synapses (38–40). So far, most researches on neurotransmitter receptors have focused on their regulation of cognitive, emotional, and CNS diseases (38–40); little is known about their functions in tumors. Dopamine receptor 1 (DRD1) was relatively widely studied and identified as a potential tumor suppressor gene among the neurotransmitter receptors. Studies have shown that DRD1 has abnormal expression in various tumors, including breast cancer, CNS tumors, gastrointestinal tumors, etc. (41, 42). Activation of DRD1 could inhibit proliferation and migration of breast cancer and promote cell apoptosis and autophagy (43, 44). The DRD1 agonist SKF38393 significantly reduced the cell viability of MCF7, an estrogen-receptor-positive breast cancer cell line, more so than tamoxifen, the first-line agent for the treatment of hormone-dependent metastatic breast cancer (14).

HTR6 is 5-hydroxytryptamine receptor 6, first identified and cloned in 1993 (45). HTR6 is conjugated with Gsα protein, and the activation of HTR6 can promote the synthesis of secondary messenger cAMP (45). This receptor is highly expressed in CNS and involved in regulating cognition, feeding, emotional state, and epilepsy, and is closely associated with schizophrenia and Alzheimer’s disease (46, 47). In 2016, Jinhua Xu’s team found that the expression of HTR6 was decreased in colon cancer, and the lower expression was closely related to tumor recurrence (48). Therefore, combined with previous studies and our findings, HTR6 is more likely to play as a tumor suppressor in tumor recurrence. Noticeably, according to the NextPharma database, two antipsychotic drugs that target HTR6 are currently in preclinical and phase I clinical studies, highlighting the importance of elucidating the role of HTR6 in tumors.

In our previous study, we found that sertindole, an antipsychotic drug, has inhibitory activity on breast cancer, and the activation of HTR6 could rescue the loss of proliferation of cells (49). This result suggested that HTR6 activity might be positively correlated with the proliferation of breast cancer cells. In the immunohistochemical detection of HTR6 in breast cancer patients, compared with normal breast tissue and para-cancerous tissues, the expression of HTR6 was increased in breast cancer *in situ* but decreased in invasive breast cancer, and almost no expression was found in distal and lymph metastases, exhibiting a wave trend during the development of breast cancer. Combined with the results of this study, we speculated that the role of HTR6 in breast cancer might have two sides, playing as a promoter for tumor proliferation but suppressive for tumor progression and recurrence. Further functional studies are needed to verify the functions of HTR6 in different phases of breast cancer.

Interestingly, we discovered a close expression correlation between HTR6 and immune response-related genes, including checkpoint genes, immune stimulators, immune receptors, and chemokines (**Figure 6**). In fact, a large number of studies have reported the regulatory relationship between serotonin (as the ligand of HTR6) and immune system. For example, taking the selective serotonin reuptake inhibitors (SSRIs) can cause a decrease in pro-inflammatory cytokines and T helper cell 17 cells (Th17), exhibiting an anti-inflammatory effect (50). On the other hand, in **Figures 3** and **4**, we found that HTR6 expression was associated with changes in phosphorylation of MAPK pathway, JUN pathway, and megakaryocyte differentiation pathway. As is well known, MAPK pathway and JUN pathway are highly involved in the induction of inflammation (51, 52). Therefore, we speculate that the co-expression between HTR6 and the immune reaction-related genes may be mediated by these two pathways.

In conclusion, our study clarified a new function of HTR6 in breast cancer, uncovered its role in regulating the immune microenvironment, and revealed its relevant pathways and upstream regulator. The findings of this study laid a theoretical basis for the functions of neurotransmitter receptors in tumors, improved our understanding of the pathogenesis of breast cancer, and provided evidence for tumor prevention and personalized medicine in patients with mental illness.

## Data Availability Statement

The original contributions presented in the study are included in the article/**Supplementary Material**. Further inquiries can be directed to the corresponding authors.

## Ethics Statement

The studies involving human participants were reviewed and approved by The Ethics Committee of Taizhou Hospital. The patients/participants provided their written informed consent to participate in this study.

## Author Contributions

WZ, YD, YX, and DT: conceived the study and study design. WZ, LL, and JL: data extraction, elaboration and manuscript writing. BY, WC, and YC: patient clinical information collection and experiment performing; HY, FZ, and LY: data interpretation and discussion. All authors: manuscript editing. All authors read and approved the final manuscript.

## Funding

This study was supported by the National Natural Science Foundation of China (No.82003172), Shenzhen Fund for Guangdong Provincial High-level Clinical Key Specialties (No.SZGSP001), the Postdoctoral Science Foundation of China (No.2020M673065), the Guangdong Basic and Applied Basic Research Foundation (No.2019A1515111138) and the science and technology plan of Shenzhen (No.JCYJ20180306140810282).

## Conflict of Interest

Author WZ were employed by company HaploX Biotechnology.

The remaining authors declare that the research was conducted in the absence of any commercial or financial relationships that could be construed as a potential conflict of interest.

## Publisher’s Note

All claims expressed in this article are solely those of the authors and do not necessarily represent those of their affiliated organizations, or those of the publisher, the editors and the reviewers. Any product that may be evaluated in this article, or claim that may be made by its manufacturer, is not guaranteed or endorsed by the publisher.

## Supplementary Material

Supplementary Table 1 Clinical information, immunohistochemical staining intensity and staining proportion of the 44 breast cancer patients.

Supplementary Table 2 All phosphorylation sites associated with HTR6 mRNA expression.
