# Supplementary material for: Systematic Analysis of Neurotransmitter Receptors in Human Breast Cancer Reveals a Strong Association With Outcome and Uncovers HTR6 as a Survival-Associated Gene Potentially Regulating the Immune Microenvironment
Source: Front Immunol. 2022 Mar 10;13:756928. doi: 10.3389/fimmu.2022.756928 (PMC8960964; doi:10.3389/fimmu.2022.756928)
Supplement: Supplementary file 3 [file Image_1.pdf]

Supplementary Figure S1

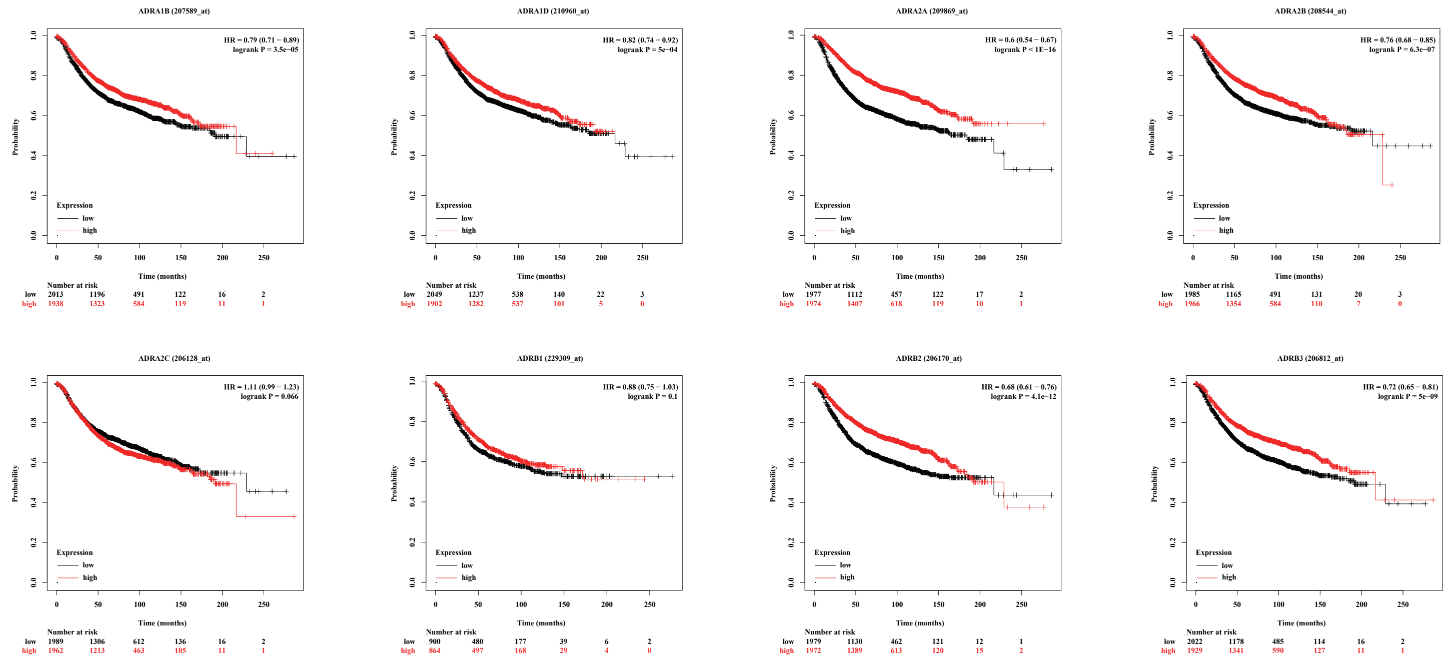

Supplementary Figure S1 The analysis of relapse-free survival of adrenergic receptors in breast cancer patients.

Supplementary Figure S2

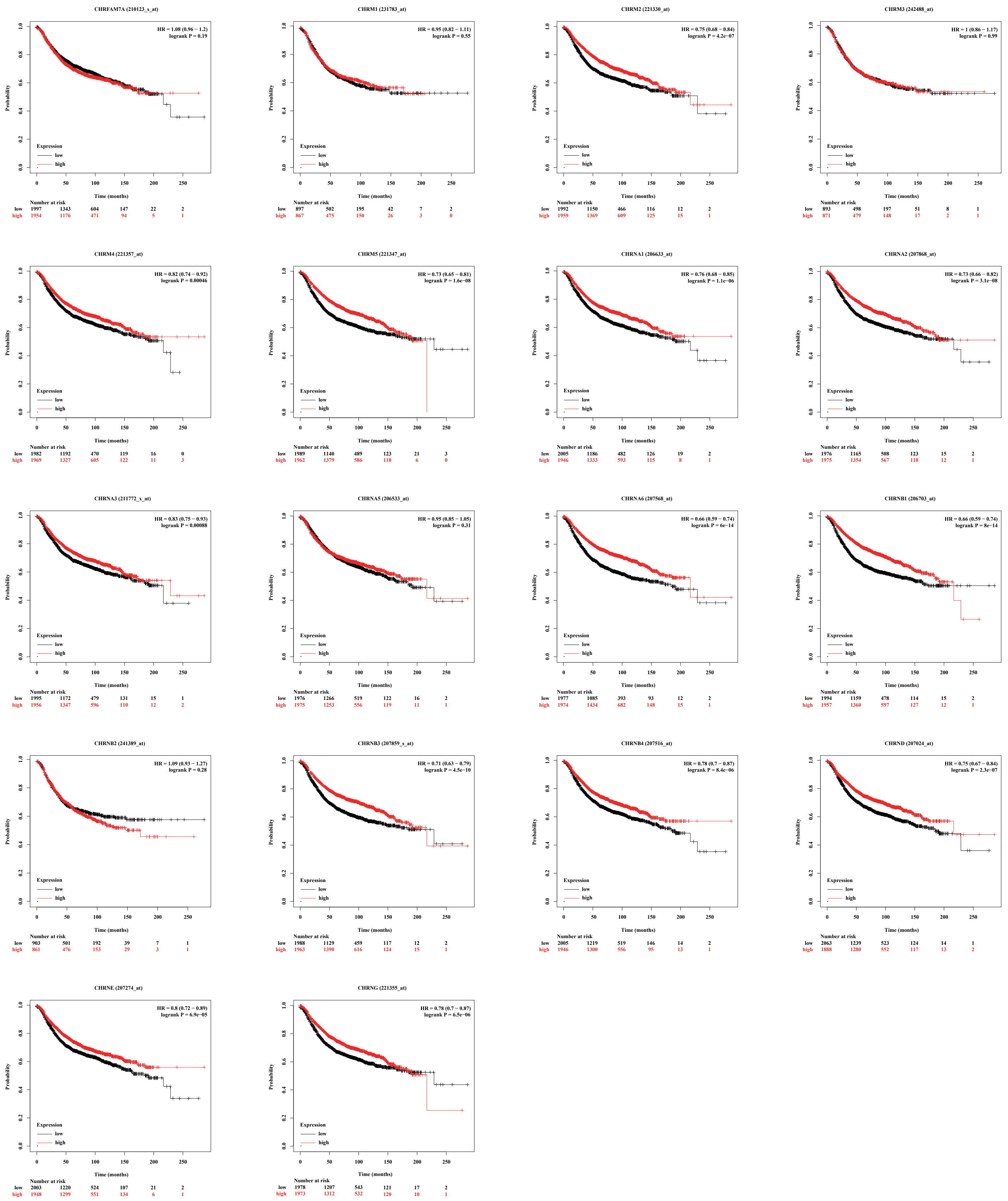

Supplementary Figure S2 The analysis of relapse-free survival of cholinergic receptors in breast cancer patients.

Supplementary Figure S3

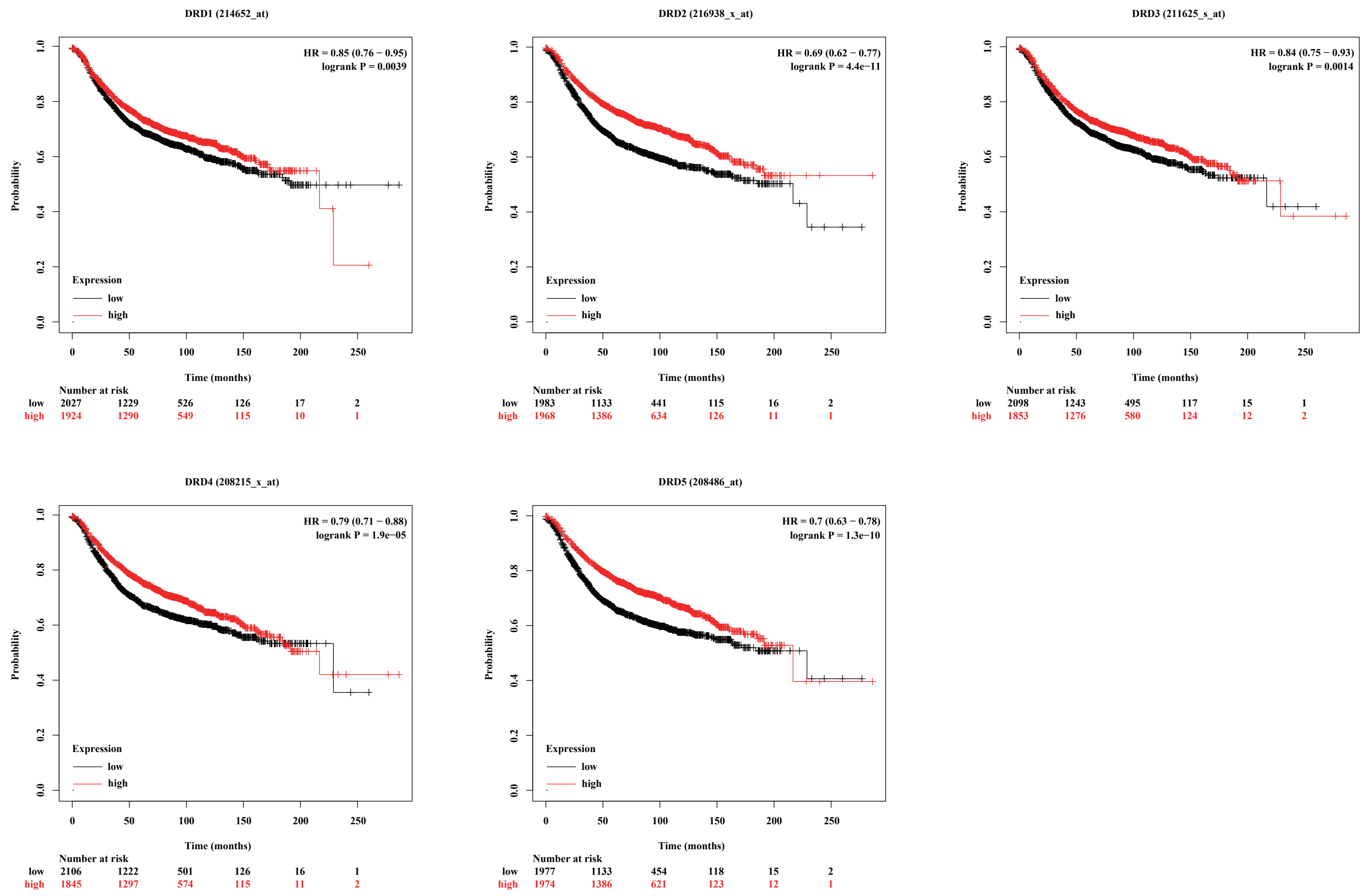

Supplementary Figure S3 The analysis of relapse-free survival of dopamine receptors in breast cancer patients.

Supplementary Figure S4

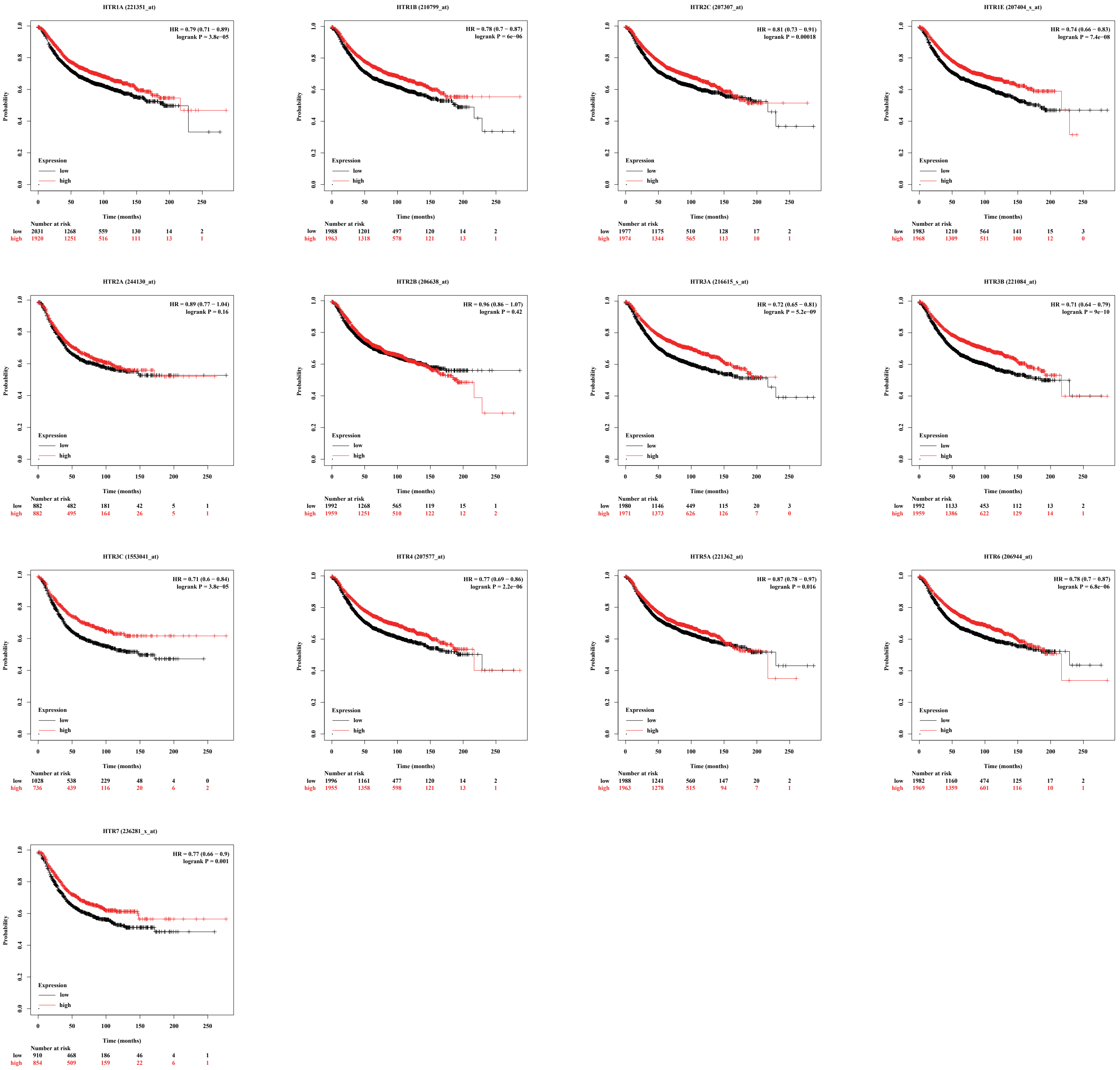

Supplementary Figure S4 The analysis of relapse-free survival of serotonin receptors in breast cancer patients.

Supplementary Figure S5

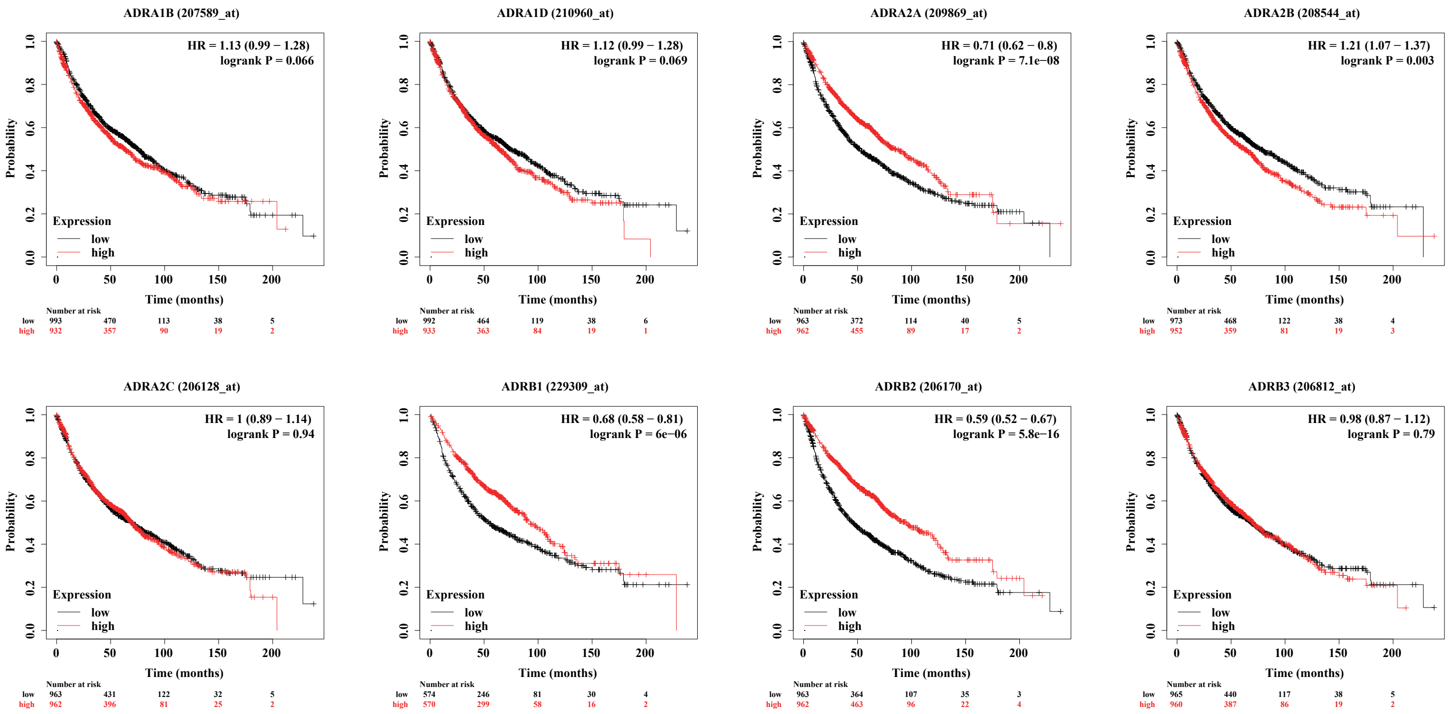

Supplementary Figure S5 The analysis of overall survival of adrenergic receptors in lung cancer patients.

Supplementary Figure S6

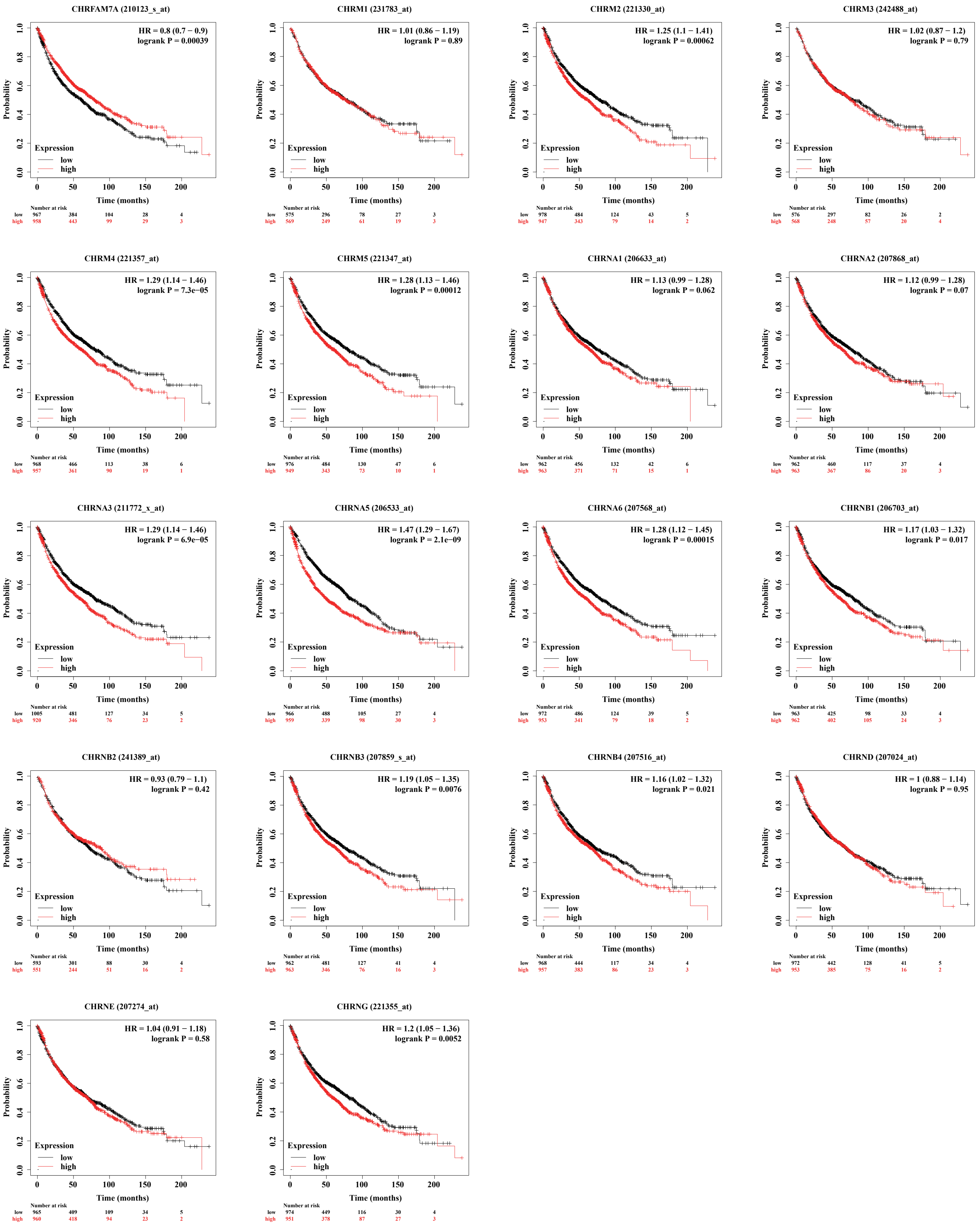

Supplementary Figure S6 The analysis of overall survival of cholinergic receptors in lung cancer patients.

Supplementary Figure S7

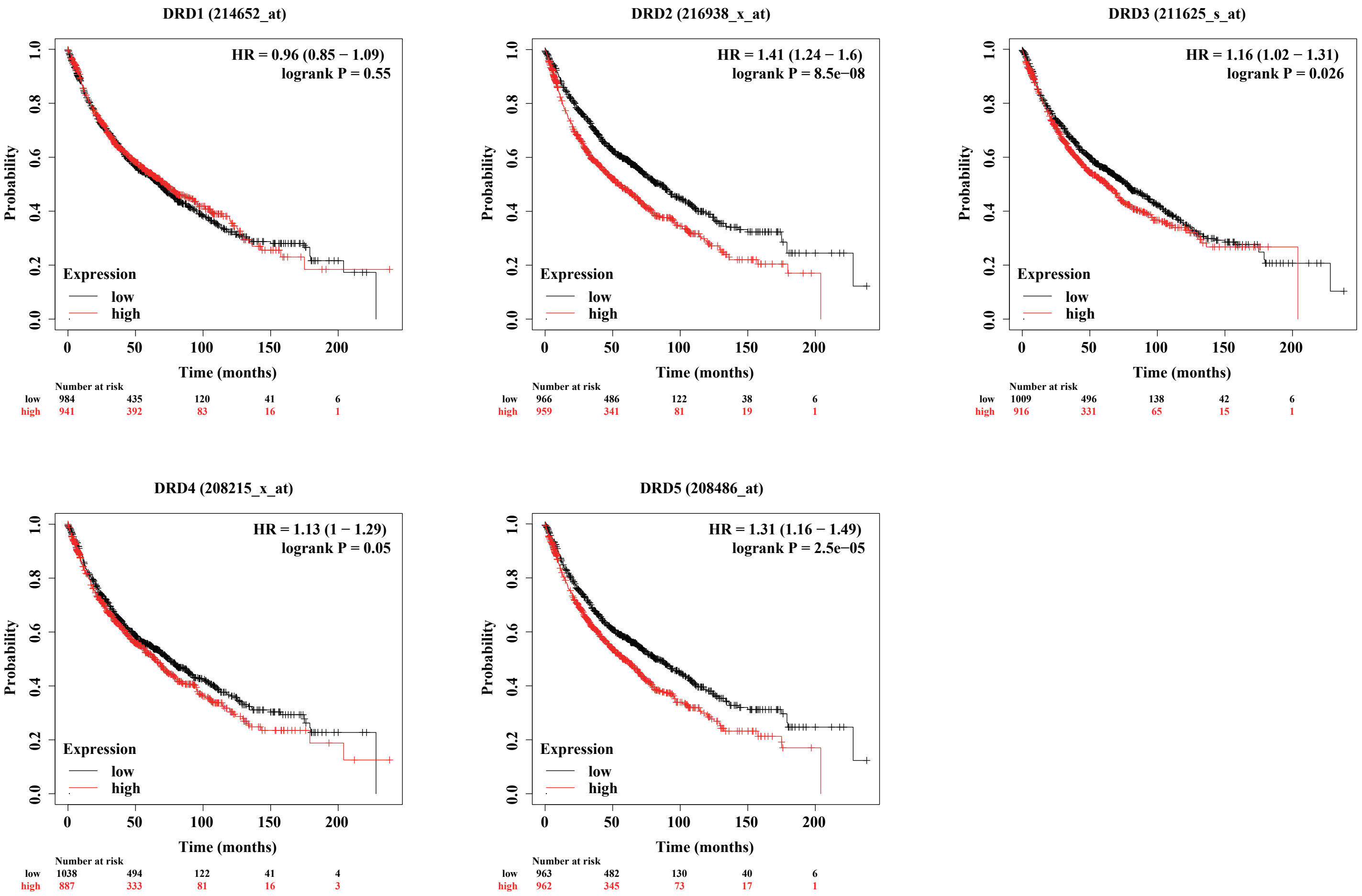

Supplementary Figure S7 The analysis of overall survival of dopamine receptors in lung cancer patients.

Supplementary Figure S8

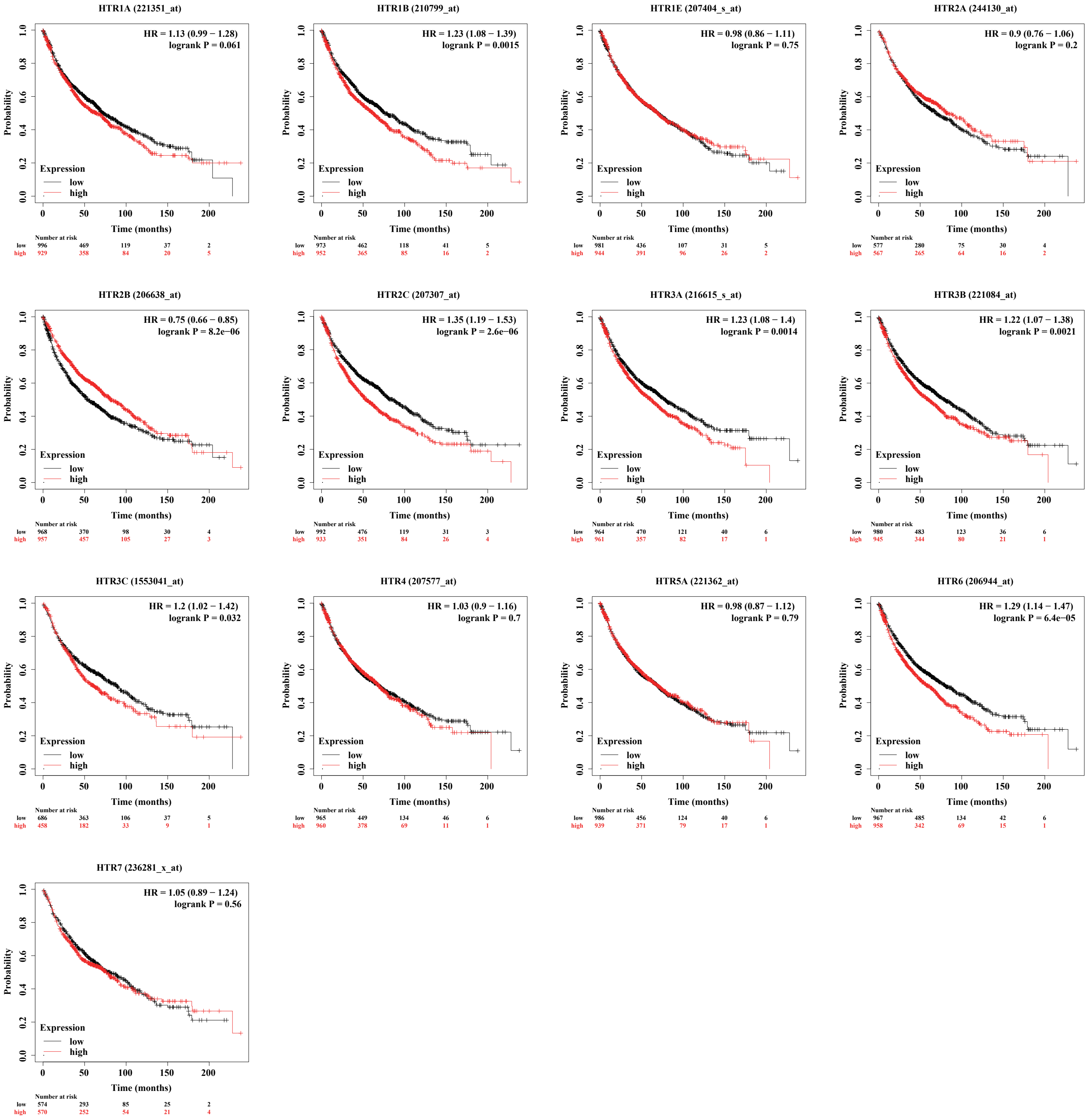

Supplementary Figure S8 The analysis of overall survival of serotonin receptors in lung cancer patients.
